# A nomogram for predicting cancer-specific survival in patients with locally advanced unresectable esophageal cancer: development and validation study

**DOI:** 10.3389/fimmu.2025.1524439

**Published:** 2025-02-14

**Authors:** Liangyun Xie, Yafei Zhang, Xiedong Niu, Xiaomei Jiang, Yuan Kang, Xinyue Diao, Jinhai Fang, Yilin Yu, Jun Yao

**Affiliations:** ^1^ The First Affiliated Hospital, and College of Clinical Medicine of Henan University of Science and Technology, Luoyang, China; ^2^ Affiliated Tangshan Gongren Hospital, North China University of Science and Technology, Tangshan, China

**Keywords:** locally advanced esophageal cancer, SEER, cancer-specific survival (CSS), prognostic nomogram, LASSO regression, immune microenvironment

## Abstract

**Background:**

Immunotherapy research for esophageal cancer is progressing rapidly, particularly for locally advanced unresectable cases. Despite these advances, the prognosis remains poor, and traditional staging systems like AJCC inadequately predict outcomes. This study aims to develop and validate a nomogram to predict cancer-specific survival (CSS) in these patients.

**Methods:**

Clinicopathological and survival data for patients diagnosed between 2010 and 2021 were extracted from the Surveillance, Epidemiology, and End Results (SEER) database. Patients were divided into a training cohort (70%) and a validation cohort (30%). Prognostic factors were identified using the Least Absolute Shrinkage and Selection Operator (LASSO) regression. A nomogram was constructed based on the training cohort and evaluated using the concordance index (C-index), net reclassification improvement (NRI), integrated discrimination improvement (IDI), calibration plots, and area under the receiver operating characteristic curve (AUC). Kaplan-Meier survival curves were used to validate the prognostic factors.

**Results:**

The study included 4,258 patients, and LASSO-Cox regression identified 10 prognostic factors: age, marital status, tumor location, tumor size, pathological grade, T stage, American Joint Committee on Cancer (AJCC) stage, SEER stage, chemotherapy, and radiotherapy. The nomogram achieved a C-index of 0.660 (training set) and 0.653 (validation set), and 1-, 3-, and 5-year AUC values exceeded 0.65. Calibration curves showed a good fit, and decision curve analysis (DCA), IDI, and NRI indicated that the nomogram outperformed traditional AJCC staging in predicting prognosis.

**Conclusions:**

We developed and validated an effective nomogram model for predicting CSS in patients with locally advanced unresectable esophageal cancer. This model demonstrated significantly superior predictive performance compared to the traditional AJCC staging system. Future research should focus on integrating emerging biomarkers, such as PD-L1 expression and tumor mutational burden (TMB), into prognostic models to enhance their predictive accuracy and adapt to the evolving landscape of immunotherapy in esophageal cancer management.

## 1 Introduction

Esophageal cancer is the eleventh most common malignancy worldwide and the seventh leading cause of cancer-related death. In 2022, it was estimated that there were 511,000 new cases and 445,000 deaths from esophageal cancer globally, accounting for approximately 5.6% of all cancer-related deaths. The incidence and mortality rates of esophageal cancer are significantly higher in men, roughly three times those in women (1). For patients with early-detected esophageal cancer, the disease is typically localized, making surgical treatment the preferred approach. However, early-stage esophageal cancer often presents with subtle or atypical symptoms, resulting in many patients being diagnosed at intermediate or advanced stages and missing the chance for surgical intervention (2). Locally advanced esophageal cancer (LAEC) refers to tumors that have invaded the deeper layers of the esophageal wall or adjacent structures and may involve regional lymph node metastasis but without distant metastasis (M0). According to the American Joint Committee on Cancer (AJCC) staging system, LAEC is generally defined as T2 or higher with or without lymph node involvement (N+) (3). For these patients, radiochemotherapy is the primary treatment modality, but the prognosis remains poor, with a five-year survival rate of only 20% (4).

The TNM staging system, widely regarded as the gold standard for tumor prognosis assessment, categorizes esophageal cancer based on tumor depth (T stage), lymph node involvement (N stage), and distant metastasis (M stage) (5). However, it has limitations due to its exclusive focus on tumor biology, neglecting patient-specific factors like age, gender, marital status, and pathological grade. Consequently, it fails to provide a comprehensive prognosis for esophageal cancer patients undergoing multimodal treatment, especially for those with locally advanced disease who experience recurrence or progression despite comprehensive therapies, with a median survival of 4 to 28 months (6, 7). This highlights the need for more comprehensive predictive tools. To address this, researchers are exploring methods that integrate a broader range of clinical data, including tumor biology and patient characteristics, for more accurate prognosis prediction. One such tool is the nomogram, which consolidates multiple prognostic factors into a graphical representation, supporting personalized medicine (8). This approach enhances prognosis prediction and clinical decision-making, leading to more precise treatment plans for patients with locally advanced esophageal cancer.

The National Cancer Institute’s (NCI) Surveillance, Epidemiology, and End Results (SEER) database is a crucial national cancer dataset that encompasses detailed demographic and clinical data for approximately one-third of the U.S. population (9). It has become a key authoritative source for providing information on cancer incidence and survival in the U.S. Initially launched in 1973, SEER began with data from seven primary tumor registries and has since expanded to cover 22 registries, representing 48% of cancer cases nationwide (10). The SEER program stands as a gold standard for population-level cancer data collection, providing invaluable insights into incidence, survival, and mortality by histopathologic and molecular subtypes (11). Over recent years, SEER has evolved significantly to adapt to the modern era of precision oncology. The database now captures molecular data, treatment details, and longitudinal outcomes, addressing gaps in real-world evidence and enabling better assessment of cancer care’s effectiveness in diverse populations (12). This comprehensive approach bridges the gap between clinical trial findings and general population outcomes, highlighting its pivotal role in supporting both clinical research and population-level decision-making.

This study aims to utilize the SEER database to evaluate potential risk factors affecting the prognosis of patients with locally advanced unresectable esophageal cancer, to develop and validate a nomogram integrating clinically relevant prognostic factors for predicting tumor-specific survival in patients undergoing radical esophagectomy, and to compare its predictive performance with the traditional AJCC staging system.

## 2 Methods

### 2.1 Data sources and patient selection

This study utilized the April 2024 release of the SEER database, annually updated since 1973, to analyze cancer incidence, prevalence, and mortality trends. Data were sourced from SEER*Stat version 8.4.3, encompassing cancer registries from 17 U.S. regions (2000-2021), representing about 30% of the U.S. population. The dataset provided detailed patient demographics, tumor characteristics, treatment details, and survival status. As SEER data are publicly available and anonymized, ethical review and informed consent were not required. Research adhered to the 1964 Declaration of Helsinki and its amendments.

**Figure 1:**
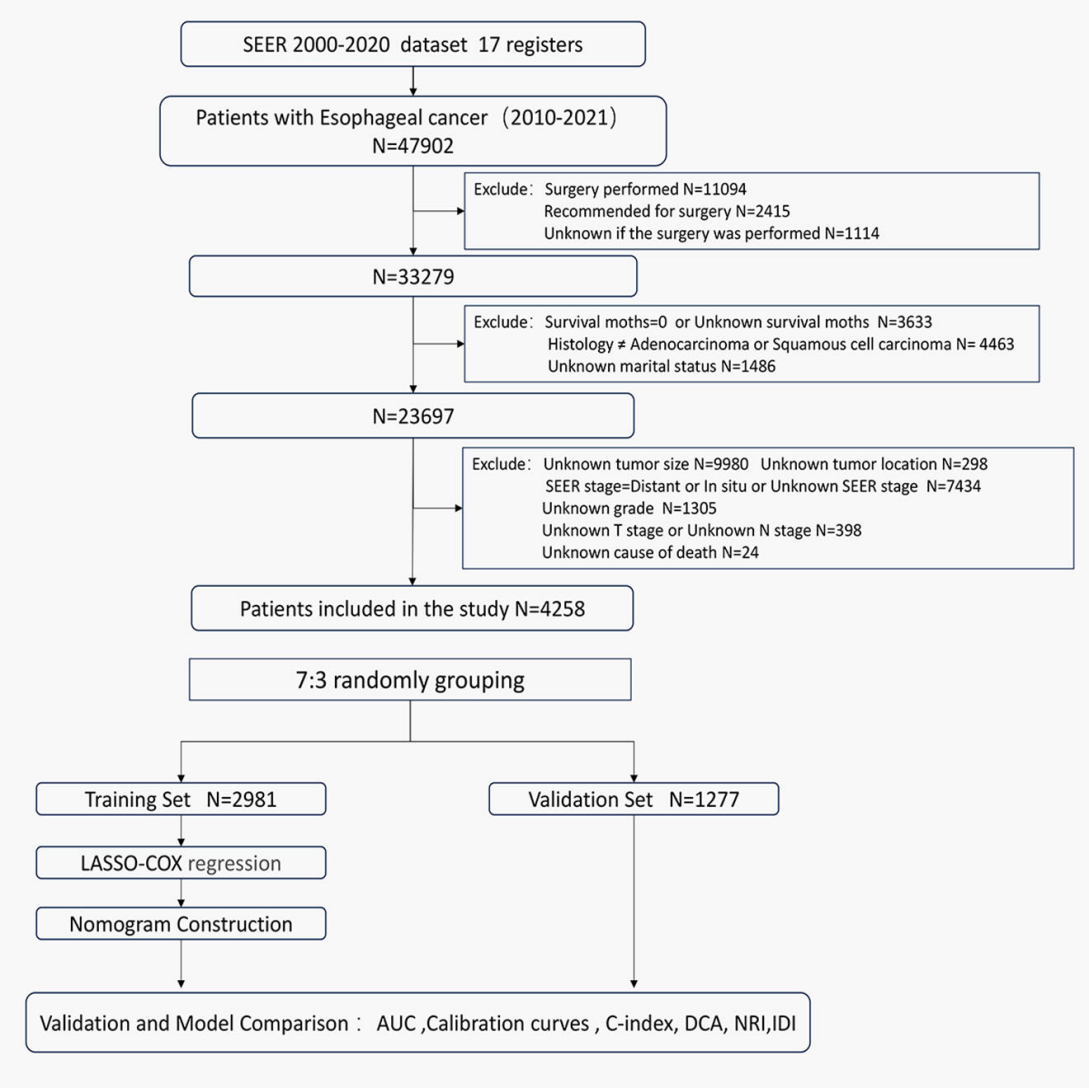
Flowchart of the patients screening process.

Owing to database limitations, the scope of this study was confined to sample data from 2000 to 2021. Initially, we selected patients with cancers of the esophagus according to the third edition of the International Classification of Diseases for Oncology (ICD-O-3: C15.0–C15.9). We further refined our selection to include patients diagnosed with locally advanced esophageal cancer between 2010 and 2021, based on the SEER historical staging system. The SEER staging system categorizes cancer into four stages: *in situ*, local, regional, and distant disease. Local disease is defined as a tumor confined to a local anatomical region of the esophagus without the involvement of the esophageal serosa or regional lymph nodes (T1-2N0M0). Regional disease refers to tumors that have extended within the regional anatomical area but have not metastasized distantly (T3-4aN0M0 or T1-4aN1-3M0). Distant disease indicates the presence of distant metastases (10, 13). Therefore, for this study, both local and regional diseases were categorized as locally advanced esophageal cancers. The exclusion criteria were as follows: (1) patients with carcinoma *in situ* or distant metastasis; (2) patients for whom surgery was recommended, who had undergone surgery, or whose surgical status was unknown; (3) patients with a survival time of 0 or unknown, or with a follow-up time of 0; (4) patients with pathological types other than esophageal squamous cell carcinoma or adenocarcinoma; (5) patients of unknown race, marital status, cause of death, tumor location, tumor size, grade, T stage, N stage, or AJCC clinical stage. The flowchart of the study population selection process is shown in **Figure 1**.

The extracted information included: gender, age, race, marital status, regional income level, tumor location, tumor size, pathological type, pathological grade, T stage, N stage, AJCC clinical stage, SEER historical stage, radiation and chemotherapy status, cause of death, survival time, and survival status.

### 2.2 Study outcomes

The primary endpoint of this study is cancer-specific survival (CSS), defined as the time from esophageal cancer diagnosis to death specifically attributed to the disease. Survival time was calculated from diagnosis to death or last follow-up, excluding participants with unknown death causes, unknown survival times, or zero survival time. Death causes were classified based on the SEER Cause of Death Classification. SEER database’s “Vital Status” and “Survival months” data were used to compute CSS for patients.

### 2.3 Statistical analysis

The study cohort was randomly split into training (70%) and internal validation (30%) sets. X-tile software (Yale University Rimm Lab, New Haven, CT, USA) was used to determine optimal cut-off points for continuous variables by maximizing the separation of Kaplan-Meier survival curves, ensuring the best predictive performance. Basic clinical characteristics were compared using the χ² test. Least Absolute Shrinkage and Selection Operator (LASSO) regression identified esophageal cancer prognosis factors in the training set. LASSO regression was employed to select prognostic factors by penalizing regression coefficients and shrinking some of them to zero, thereby addressing multicollinearity and reducing overfitting in the model. Subsequent Cox regression was conducted to quantify the hazard ratios for each variable identified by LASSO regression, ensuring that the selected factors were meaningfully associated with CSS and providing interpretable estimates for clinical application. Based on these selected variables, a Cox regression model was developed, and a nomogram was constructed to predict each patient’s cancer-specific survival rates at 1 year, 3 years, and 5 years post-diagnosis. Receiver Operating Characteristic (ROC) curves, Area Under the Curve (AUC), and Concordance Index (C-index) were used to assess predictive accuracy, with thresholds for interpretation provided. AUC and C-index values range from 0.5 to 1, where 0.5 indicates no predictive ability and 1 indicates perfect concordance. Typically, an AUC and C-index below 0.6 represents low discrimination, 0.6 to 0.75 represents moderate discrimination, and above 0.75 represents high discrimination (14). Calibration curves validated model performance. The nomogram’s accuracy was compared to the American Joint Committee on Cancer (AJCC) staging system using Net Reclassification Improvement (NRI) and Integrated Discrimination Improvement (IDI) values. Higher AUC values in time-dependent ROC curves indicated superior prognostic accuracy. Variables were scored and summed to predict CSS, categorizing patients into low, intermediate, and high-risk subgroups using X-tile-determined cutoff points. Kaplan-Meier (K-M) survival curves were plotted for both sets. Decision Curve Analysis (DCA) evaluated the nomogram’s clinical utility. All analyses were conducted using R software (R Foundation for Statistical Computing, Vienna, Austria), with statistical significance set at p<0.05.

## 3 Results

### 3.1 Baseline characteristics and survival

A total of 47,902 patients diagnosed with esophageal cancer between 2010 and 2021 were extracted from the SEER database. After applying exclusion criteria, 43,644 cases were removed, leaving 4,258 patients with locally advanced unresectable esophageal cancer for further analysis. These patients were randomly divided into a training set (2,981 patients) and a validation set (1,277 patients) at a 7:3 ratio. Optimal cutoff values for age and tumor size were determined using X-tile software, and patients were stratified based on these thresholds (see **Supplementary Figure S1**). In the overall cohort, males were more prevalent, comprising 72.4% of the sample, with a male-to-female ratio of 2.63:1. The majority of patients were White, accounting for 82% of the cohort. The median age at diagnosis was 71 years. Among the patients, 420 (9.9%) were over 85 years old, 1,223 (28.7%) were between 75 and 85 years old, and 2,615 (61.4%) were under 75 years old. In the training set, the median follow-up time was 44 months (range: 1-143 months), with 1-year, 3-year, and 5-year cancer-specific survival rates of 57.4%, 30.7%, and 23.1%, respectively. The median CSS was 16 months. In the validation set, the median follow-up time was 42 months (range: 1-125 months), with 1-year, 3-year, and 5-year CSS rates of 58.0%, 29.0%, and 23.7%, respectively, and a median CSS of 16 months. Detailed clinical characteristics of the patients are presented in **Table 1**.

**Table 1:** Demographics and characteristics of patients in training and validation sets.

Variables	Training data set (n=2981)	Validation data set (n=1277)	p-value
Sex			0.91
Female	819 (27.5%)	353 (27.6%)	
Male	2,162 (72.5%)	924 (72.4%)	
Age			0.629
<=75	1,817 (61.0%)	798 (62.5%)	
75-85	868 (29.1%)	355 (27.8%)	
>85	296 (9.9%)	124 (9.7%)	
Race			0.118
White	2,421 (81.2%)	1,071 (83.9%)	
Black	345 (11.6%)	127 (9.9%)	
Others	215 (7.2%)	79 (6.2%)	
Marital			0.298
Married	1,653 (55.5%)	686 (53.7%)	
Unmarried and others	1,328 (44.5%)	591 (46.3%)	
Income.			0.034
<=70000	1,040 (34.9%)	489 (38.3%)	
>70000	1,941 (65.1%)	788 (61.7%)	
Tumor.Location			0.532
Lower	1,631 (54.7%)	723 (56.6%)	
Middle	779 (26.1%)	307 (24.0%)	
Upper	444 (14.9%)	194 (15.2%)	
Overlapping	127 (4.3%)	53 (4.2%)	
Tumor.size			0.613
<=30	887 (29.8%)	393 (30.8%)	
30-76	1,570 (52.7%)	674 (52.8%)	
>76	524 (17.6%)	210 (16.4%)	
Histology			0.495
Squamous cell carcinoma	1,465 (49.1%)	613 (48.0%)	
Adenocarcinoma	1,516 (50.9%)	664 (52.0%)	
Grade			0.62
GradeI-II	1,640 (55.0%)	692 (54.2%)	
GradeIII-IV	1,341 (45.0%)	585 (45.8%)	
T.stage			0.493
T1	710 (23.8%)	311 (24.4%)	
T2	477 (16.0%)	225 (17.6%)	
T3	1,518 (50.9%)	622 (48.7%)	
T4	276 (9.3%)	119 (9.3%)	
N.stage			0.892
N0	1,417 (47.5%)	617 (48.3%)	
N1	1,317 (44.2%)	559 (43.8%)	
N2	212 (7.1%)	89 (7.0%)	
N3	35 (1.2%)	12 (0.9%)	
AJCC.stage			0.799
I	606 (20.3%)	261 (20.4%)	
II	675 (22.6%)	305 (23.9%)	
III	1,379 (46.3%)	581 (45.5%)	
IVA	321 (10.8%)	130 (10.2%)	
SEER Summary.Stage			0.368
Regional	2,153 (72.2%)	905 (70.9%)	
Localized	828 (27.8%)	372 (29.1%)	
Chemotherapy			0.94
Yes	2,357 (79.1%)	1,011 (79.2%)	
No/Unknown	624 (20.9%)	266 (20.8%)	
Radiotherapy			0.542
Yes	2,506 (84.1%)	1,083 (84.8%)	
No/Unknown	475 (15.9%)	194 (15.2%)	

### 3.2 Selection of clinical features for the nomogram

**Figure 2:**
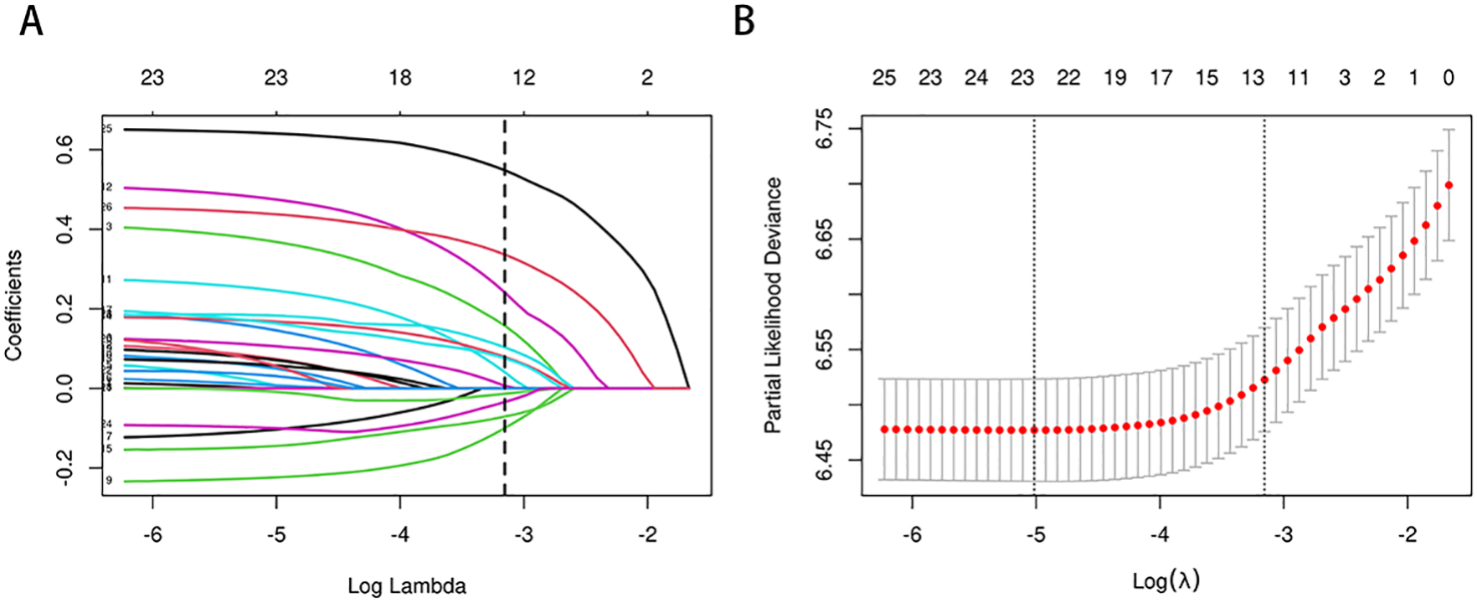
**(A, B)** Predictor selection for LASSO regression analysis. Selection of Clinicopathological Features via LASSO Coefficient Profiles **(A)**; Tuning of Regularization Parameter λ in LASSO Regression **(B)**.

In the training cohort, a univariate Cox regression analysis was conducted to evaluate the association between each potential prognostic factor and CSS in esophageal cancer patients (see **Supplementary Table S1**). The analysis identified 14 factors significantly associated with patient prognosis (P<0.05), including age, race, marital status, regional income level, tumor location, tumor size, pathological type, pathological grade, T stage, N stage, AJCC clinical stage, SEER historical stage, chemotherapy, and radiotherapy. To refine the candidate prognostic factors, we applied LASSO regression analysis. LASSO regression was utilized to select prognostic factors by applying an L1-penalty, which shrinks the coefficients of less significant variables toward zero, effectively excluding variables with limited predictive contribution. This method ensures that only the most predictive and non-redundant variables are retained for further modeling. Variables that were statistically significant in the univariate Cox analysis but had a LASSO regression coefficient of zero were excluded from the final multivariate model due to their minimal contribution to overall survival prediction. This technique reduces the number of factors by penalizing less significant variables, resulting in a more streamlined model. The LASSO regression identified 10 key prognostic factors: age, marital status, tumor location, tumor size, pathological grade, T stage, AJCC stage, SEER stage, chemotherapy, and radiotherapy. The cross-validation error plot of the LASSO regression model illustrated the model’s regularization effect. The optimal model, including the 10 selected variables, demonstrated a cross-validation error within one standard error of the minimum value (see **Figure 2**). Detailed information on the coefficients is provided in **Supplementary Table S2**.

### 3.3 Development and validation of the nomogram

**Figure 3:**
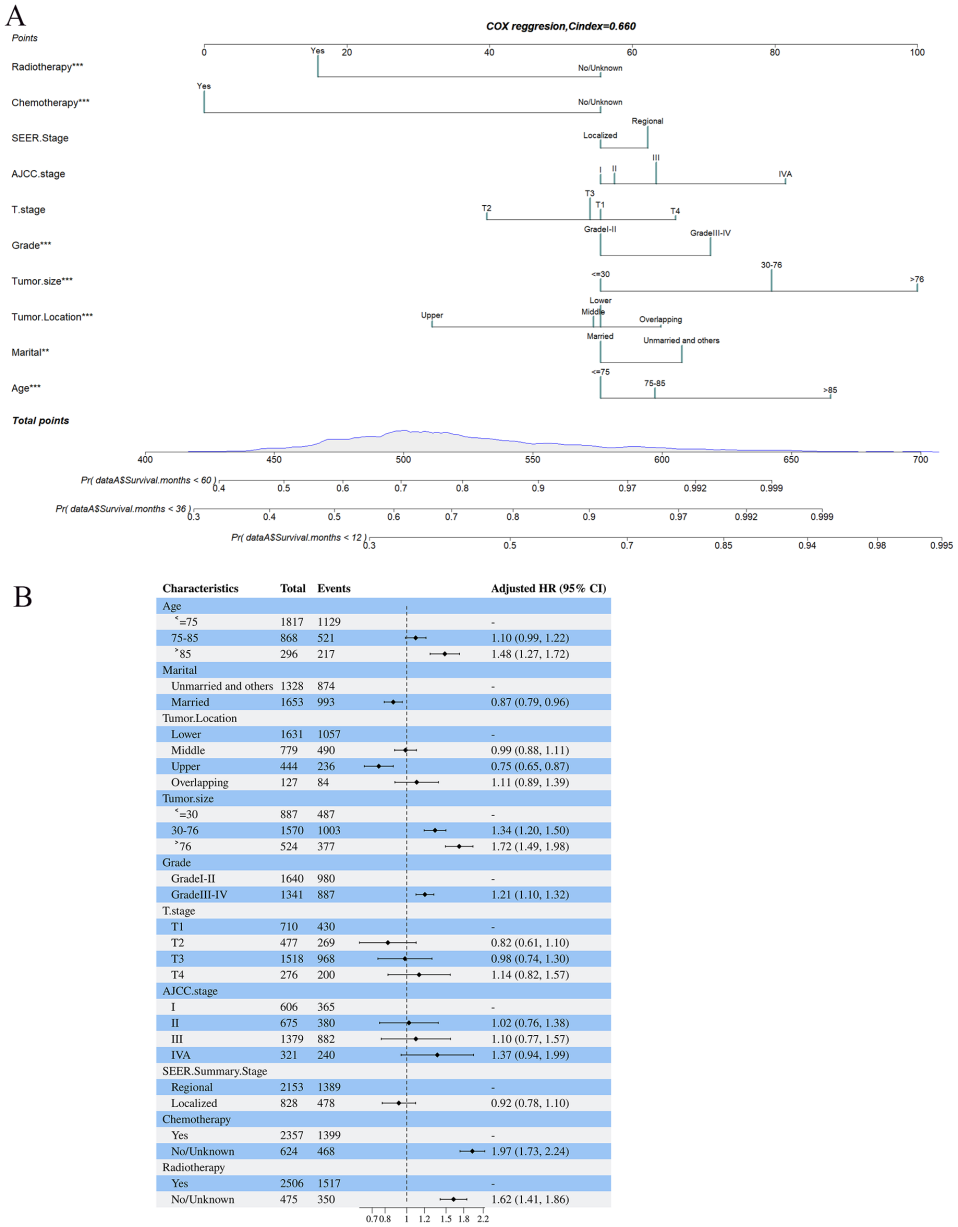
**(A, B)** Nomogram predicting cancer-specific survival based on prognostic factors developed from the training set **(A)**; Forest plots displaying the multivariable analysis of prognostic factors for Cancer-specific survival in training set **(B)**.

**Figure 4:**
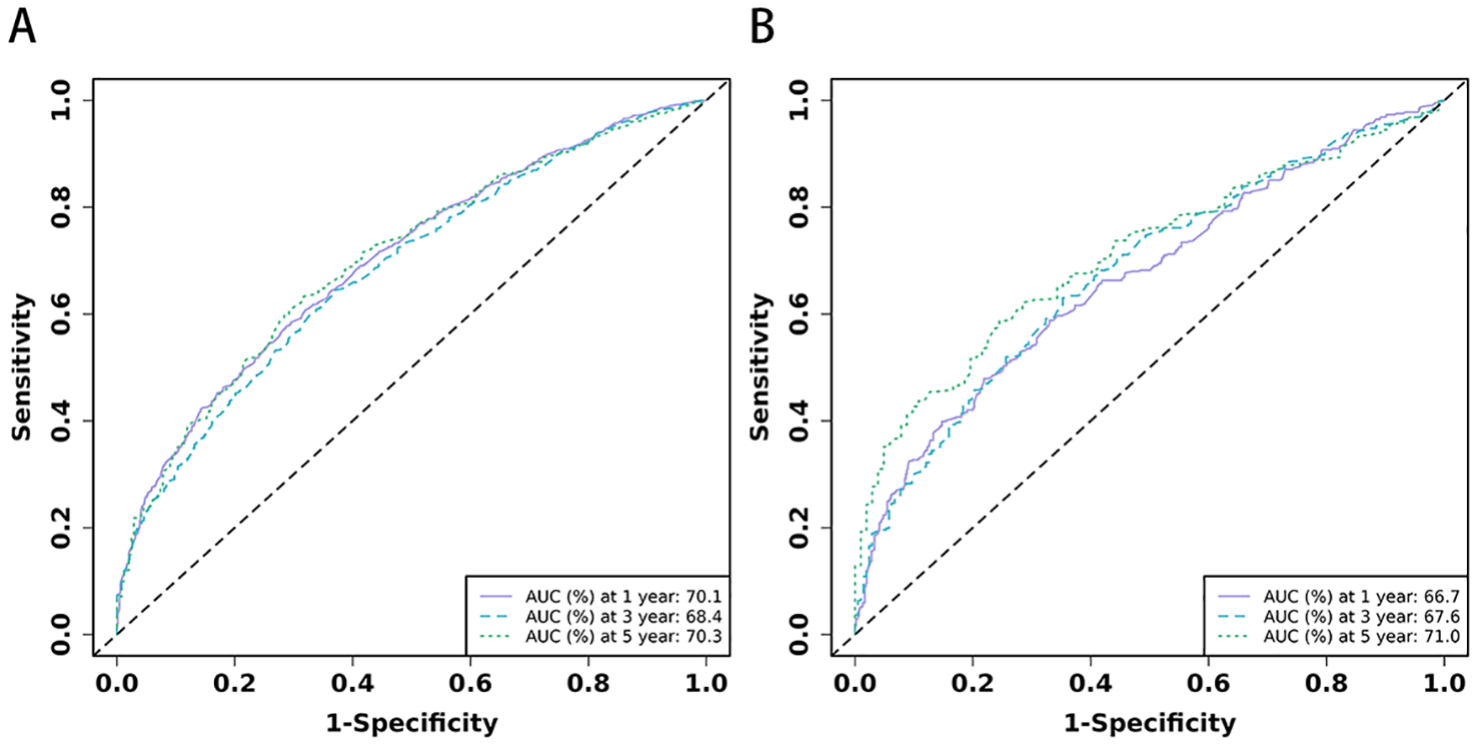
**(A, B)** The time-dependent ROC curves for nomogram. The time-dependent ROC curves 1-,3-and 5-year for nomogram in training set **(A)** and validation set **(B)**.

**Figure 5:**
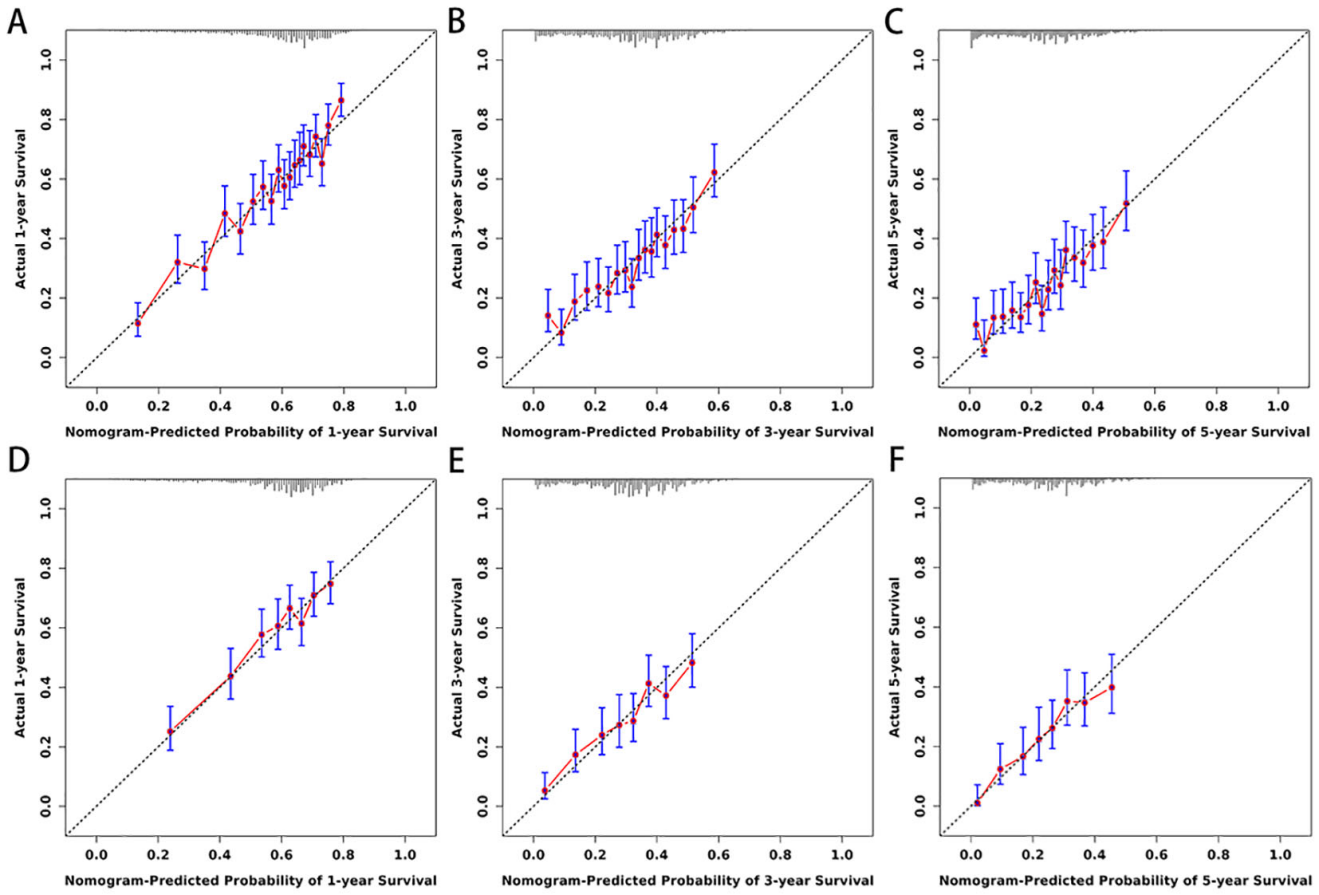
**(A–F)** Calibration curves for nomogram. The 1-**(A)**, 3-**(B)**, and 5-year **(C)** calibration curves for nomogram in training set. The 1-**(D)**, 3-**(E)**, and 5-year **(F)** calibration curves for nomogram in validation set.

**Figure 6:**
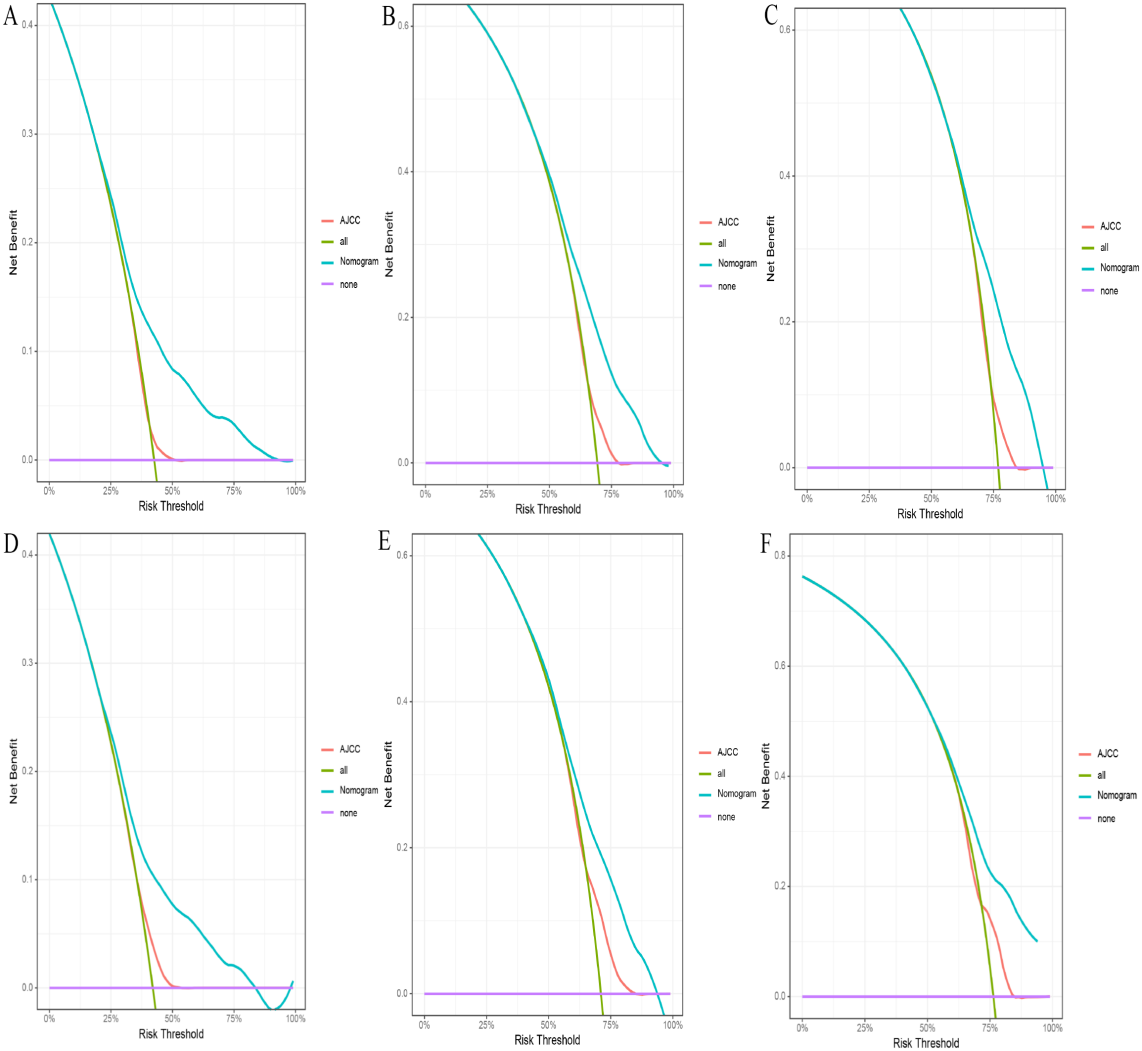
**(A-F)** The DCA for nomogram and AJCC stage. The 1-**(A)**, 3-**(B)**, and 5-year **(C)** DCA for nomogram and AJCC stage in the training set. The 1- **(D)**, 3- **(E)**, and 5-year **(F)** DCA for nomogram and AJCC stage in the validation set. DCA, Decision analysis curve.

Based on the 10 prognostic factors identified through LASSO regression, we utilized the training dataset to construct a multivariate Cox regression forest plot and a predictive nomogram for cancer-specific survival in esophageal cancer patients (see **Figure 3**). In the nomogram, each predictor’s impact on survival is represented by the length of the line associated with it. Among the predictors, chemotherapy status had the greatest impact on survival, followed by tumor size and radiation therapy status, while SEER staging had the least impact. Using the nomogram, we calculated the 1-year, 3-year, and 5-year CSS for each patient. By summing the scores for each predictor, we derived the predicted survival rates. Higher scores generally corresponded with poorer outcomes. The nomogram’s performance was assessed using the C-index. For the training set, the C-index was 0.660 (95% CI = 0.646-0.674), and for the validation set, it was 0.653 (95% CI = 0.632-0.675). The 95% confidence intervals of the C-index did not include 0.5, indicating that the predictive performance of the nomogram was statistically significant and robust. ROC curves were plotted, and the AUC values were computed for each time point. In the training set, the AUCs were 0.701, 0.684, and 0.703 for 1-year, 3-year, and 5-year CSS, respectively. In the validation set, the AUCs were 0.667, 0.676, and 0.710(see **Figure 4**). All AUC values exceeded 0.65, suggesting the model’s robust discriminative power. Calibration curves were plotted to compare predicted survival probabilities with actual outcomes. The results showed good agreement between the nomogram predictions and observed survival (see **Figure 5**). DCA indicated that the nomogram provides a valuable clinical tool for predicting CSS in patients with locally advanced unresectable esophageal cancer (see **Figure 6**). The decision curve analysis demonstrated that the nomogram adds value over the traditional AJCC staging system in clinical decision-making.

**Figure 7:**
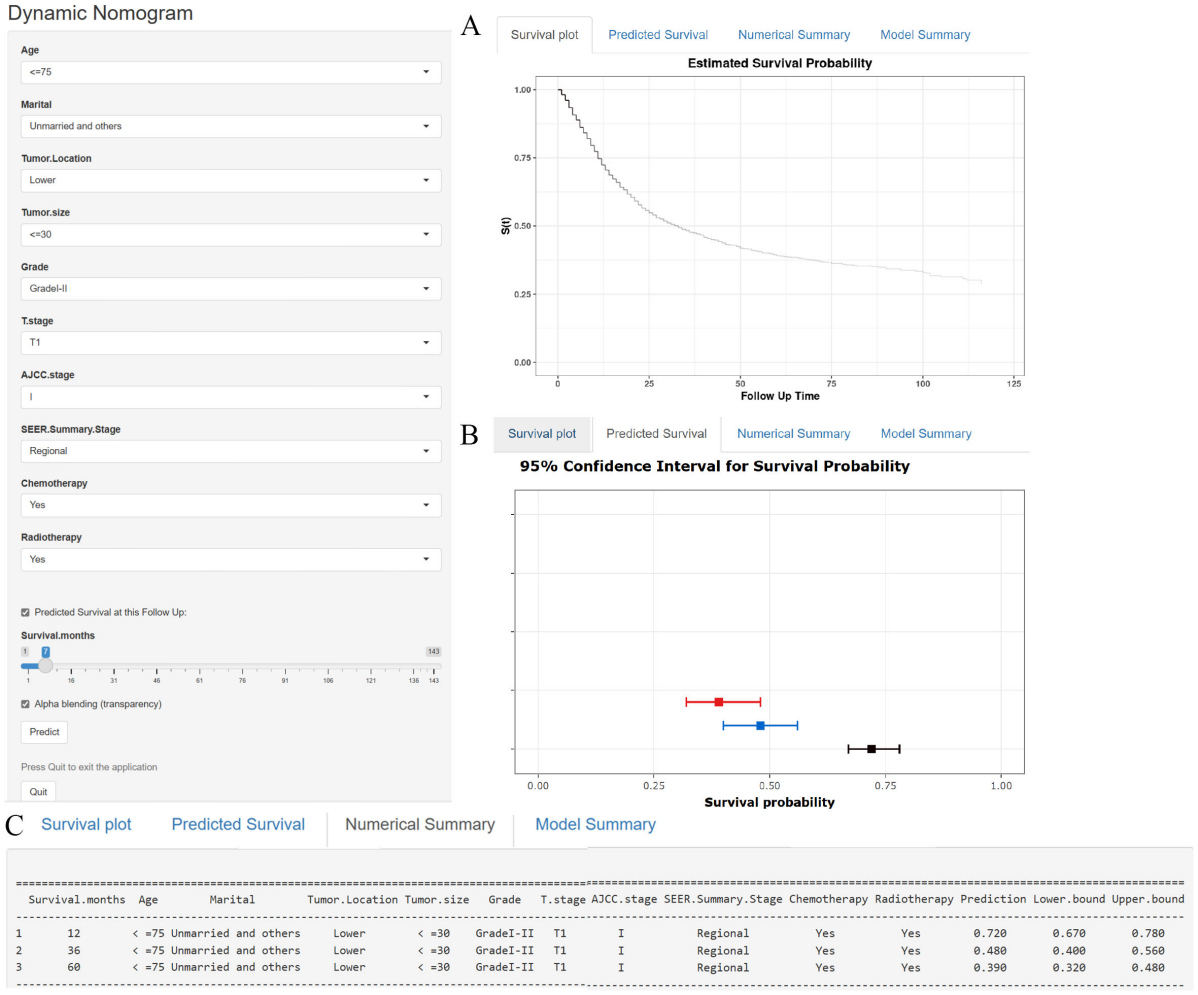
**(A–C)** A web-based nomogram for predicting cancer-specific survival in patients with locally advanced unresectable esophageal cancer. **(A)** The curve of estimated cancer-specific survival probability for those patients over time. **(B)** The 95% CI of the 1-, 3-, and 5- year cancer-specific survival probabilities for those patients. **(C)** The numerical summary of the 1-, 3-, and 5- year cancer-specific survival probabilities for those patients.

In addition to the static nomogram, a web-based dynamic nomogram tool was developed to enhance clinical usability (https://xie523205400.shinyapps.io/dynnomapp/). Created using the R Shiny package, this interactive tool integrates the 10 identified prognostic factors and allows clinicians to input patient-specific values via an intuitive interface. Key variables, such as age, tumor size, and treatment details, can be entered through dropdown menus or sliders (see **Figure 7**, left panel). A dedicated slider for “Survival months” enables users to specify the follow-up time, ensuring personalized survival predictions tailored to individual timelines. Upon entering the data, the tool generates survival predictions displayed in multiple formats for ease of interpretation, including Kaplan-Meier-like survival curves, confidence interval plots, and numerical summaries of the predicted outcomes (see **Figure 7**). This user-friendly platform facilitates real-time clinical decision-making, supporting personalized risk assessment, treatment planning, and patient counseling.

### 3.4 Comparison of the nomogram model and the 8th edition AJCC staging system

In the comparison between the 8th edition AJCC staging system and the nomogram, the ROC curve AUC values for 1-year, 3-year, and 5-year survival rates in the training cohort were 0.538, 0.567, and 0.579, respectively, and 0.554, 0.583, and 0.622 in the validation cohort, all significantly lower than those of the nomogram (see **Figure 4** and **Supplementary Figure S2**). The AJCC staging system had a C-index of 0.532 (95% CI: 0.518-0.546) in the training cohort and 0.551 (95% CI: 0.530-0.572) in the validation cohort. In the comparison between the nomogram and the AJCC staging system (see **Table 2**), the nomogram model consistently demonstrated significantly higher AUC values and C-indexes in both the training and validation cohorts. The comparison between the nomogram and the AJCC staging system showed non-overlapping 95% confidence intervals for their respective C-index values (nomogram: 0.660, 95% CI = 0.646-0.674; AJCC: 0.532, 95% CI = 0.518-0.546), confirming the nomogram’s superior predictive performance with statistical significance. The net reclassification improvement (NRI) for 1-year, 3-year, and 5-year survival rates indicated that the nomogram’s discriminatory ability improved by 16.9%, 10.5%, and 2.5% in the training cohort (all P < 0.05) and by 16.0%, 6.7%, and 6.7% in the validation cohort (all P < 0.05). Additionally, the integrated discrimination improvement (IDI) for 1-year, 3-year, and 5-year survival rates showed that the nomogram improved by 11.4%, 8.6%, and 7.6% in the training cohort, and by 9.9%, 7.4%, and 8.0% in the validation cohort (all P < 0.0001). The DCA curves (see **Figure 6**) demonstrated that the nomogram provided better predictions of 1-year, 3-year, and 5-year CSS compared to the AJCC staging system, as it resulted in greater net benefit across nearly all threshold probabilities in both the training and validation cohorts, as well as for all patients. Therefore, the nomogram is more clinically effective and accurate than the AJCC staging system in predicting CSS for patients with esophageal cancer.

**Table 2:** Comparative performance evaluation of a nomogram versus AJCC staging system for cancer-specific survival prediction.

Indicators	Training set			Validation Set		
	Nomogram	AJCC stage	Nomogram vs. AJCC stage	Nomogram	AJCC stage	Nomogram vs. AJCC stage
C-index ^a^ (95%CI^b^)	0.660 (0.646, 0.674)	0.532 (0.518, 0.546)	—	0.653 (0.632, 0.675)	0.551 ((0.530, 0.572)	—
12-months AUC ^c^	0.701	0.538	—	0.667	0.554	—
36-months AUC	0.684	0.567	—	0.676	0.583	—
60-months AUC	0.703	0.579	—	0.71	0.622	—
12-months IDI ^d^ (P-value)	—	—	11.4% (P<0.0001)	—	—	9.9% (P<0.0001)
36-months IDI (P-value)	—	—	8.6% (P<0.0001)	—	—	7.4% (P<0.0001)
60-months IDI (P-value)	—	—	7.6% (P<0.0001)	—	—	8% (P<0.0001)
12-months NRI ^e^ (P-value)	—	—	16.9% (P<0.05)	—	—	16% (P<0.05)
36-months NRI (P-value)	—	—	10.5% (P<0.05)	—	—	6.7% (P<0.05)
60-months NRI (P-value)	—	—	2.5% (P<0.05)	—	—	6.7% (P<0.05)

^a^C-index, concordance index; ^b^CI, confidence interval; ^c^AUC, area under the curve; ^d^IDI, integrated discrimination improvement.; ^e^NRI, net reclassification index; NRI or IDI>0 indicates positive improvement, suggesting that the nomogram model achieved better prediction ability than the AJCC stage. NRI or IDI<0 indicates diminished improvement, and the nomogram model’s prediction ability was less than that of the AJCC stage. NRI or IDI = 0 indicates that the nomogram model did not change.

### 3.5 Survival risk based on the nomogram

**Figure 8:**
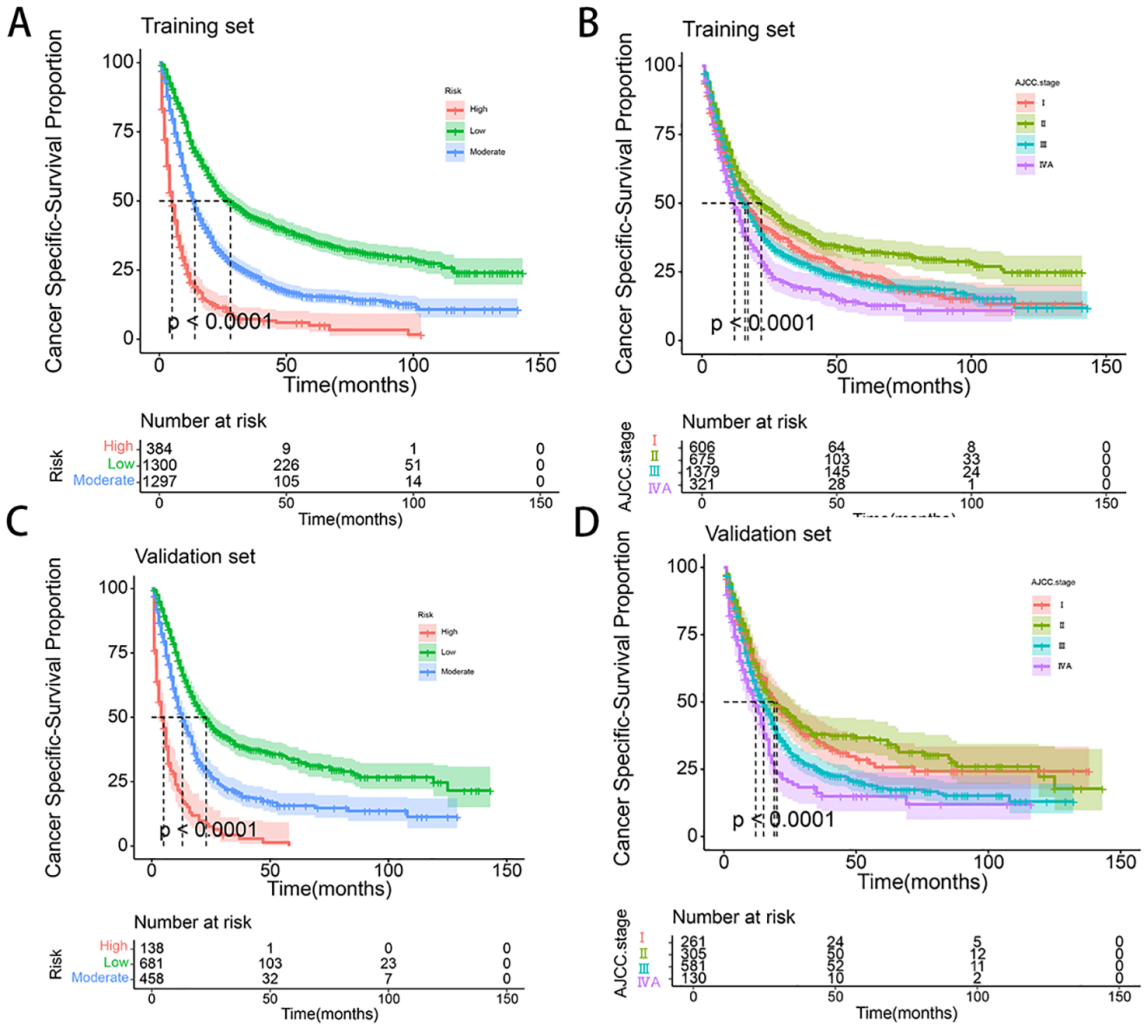
**(A-D)** Kaplan–Meier curves of CSS for risk stratification and AJCC stage in training set **(A, B)**, and validation cohort **(C, D)**.

To further validate the performance of the nomogram, we utilized X-tile software to categorize patients from both the training and validation sets into high-risk, medium-risk, and low-risk groups based on the total scores calculated from the nomogram’s CSS predictions. K-M survival curves were then generated for these risk groups. The results demonstrated significant differences in survival outcomes among the three risk categories, indicating that the nomogram model had high discriminative power and predictive accuracy (see **Figure 8**). In contrast, the AJCC staging system exhibited limited capability in risk stratification compared to the nomogram model. In the training set, the CSS curves for Stage I and Stage III patients did not show significant separation, with no statistical difference observed (HR: 1.01, 95% CI: 0.89-1.14, P=0.886). Similarly, in the validation set, the CSS curves for Stage I and Stage II patients did not exhibit significant separation, and the difference was not statistically significant (HR: 0.93, 95% CI: 0.74-1.76, P=0.498). These results highlight that the AJCC staging system, in comparison to the nomogram model developed in this study, demonstrates a lower discriminative ability and predictive accuracy for forecasting the prognosis of patients with locally advanced unresectable esophageal cancer.

## 4 Discussion

Esophageal cancer is one of the malignancies with high global incidence and mortality rates, posing a severe threat to human health and life. Histologically, esophageal squamous cell carcinoma (ESCC) and esophageal adenocarcinoma (EAC) represent the two main subtypes, with distinct etiologies and clinical features. ESCC, more common in East Asia and Africa, is strongly associated with lifestyle and environmental factors such as smoking and alcohol consumption, while EAC, prevalent in Western countries, is linked to gastroesophageal reflux disease (GERD) and obesity (1, 15–18). While the mechanisms and key risk factors of ESCC and EAC differ, both highlight the significant role of diet, lifestyle, and environmental influences in the development of esophageal cancer (19, 20). Therefore, it is crucial to identify key factors influencing the prognosis of patients with locally advanced unresectable esophageal cancer and to develop personalized prognostic models. While the TNM staging system proposed by the AJCC is widely used for assessing esophageal cancer, it primarily relies on anatomical information and may not fully capture the complexity of individualized prognosis. In recent years, nomograms have garnered significant attention as personalized tools that integrate multiple prognostic factors. These models offer more precise predictions of cancer-related survival, recurrence, and metastasis and provide valuable guidance for clinicians in formulating individualized treatment strategies.

This study included 4,258 patients diagnosed with locally advanced unresectable esophageal cancer from the SEER database between 2010 and 2021. To our knowledge, while there have been numerous predictive models developed in the field of esophageal cancer (21–27), this study represents the first research focusing specifically on developing a prognostic model for the clinical and pathological characteristics of patients with locally advanced unresectable esophageal cancer. Compared to Cox regression analysis, LASSO regression offers significant advantages in addressing issues of multicollinearity among variables. Therefore, our study utilized a LASSO-Cox regression model to develop a predictive nomogram. To construct the nomogram, we randomly divided the dataset into a training cohort (70%) and a validation cohort (30%). Analysis of the training cohort identified age, marital status, tumor location, tumor size, pathological grade, and treatment modalities (chemotherapy and radiotherapy) as significant independent prognostic factors. It is noteworthy that although the T stage, AJCC stage, and SEER stage variables had p-values greater than 0.05 in the multivariate Cox regression analysis, indicating a lack of traditional statistical significance, these clinical staging variables were still included in the prognostic model and nomogram. This inclusion is justified because these variables encapsulate tumor size, depth of invasion, lymph node involvement, and distant metastasis, reflecting disease progression and staging comprehensively. Incorporating these staging variables enhances the model’s comprehensiveness and robustness, providing a more detailed understanding of the disease state and overall patient condition. Based on extensive data from the SEER database, our study established a nomogram for predicting 1-year, 3-year, and 5-year CSS for patients with locally advanced unresectable esophageal cancer. By scoring the variables and summing the scores, we generated the nomogram and drew vertical lines corresponding to total scores to estimate CSS for 1-year, 3-year, and 5-year intervals. The validation cohort confirmed the accuracy of the nomogram, highlighting its potential value in assessing patient prognosis and guiding personalized treatment strategies. Our findings are consistent with previous research, demonstrating that the nomogram outperforms the traditional AJCC staging system in both the training and validation cohorts, achieving higher C-indexes, AUC values, and DCA scores (19, 28, 29). The nomogram stratifies patients into three risk groups based on cumulative risk scores, with increasing scores correlating with significantly reduced predicted CSS. In contrast, the AJCC staging system falls short in distinguishing between all subgroups. Future research should further validate the role of these prognostic variables to optimize the performance of the predictive model and assess its clinical application value in larger-scale trials.

Globally, the incidence and mortality rates of EC are notably higher in men compared to women. Wang et al. (30) analyzed data from 171 registries across 54 countries and observed a significantly higher incidence of esophageal cancer in men than in women. Additionally, Xu et al. (31)found a male-to-female ratio of up to 6.55:1 in a large global Phase III clinical trial involving patients with locally advanced unresectable and advanced recurrent or metastatic esophageal cancer. Our study also reported a male-to-female ratio of 2.63:1, which aligns with these findings and further corroborates the trend of higher esophageal cancer incidence in men. Several factors may contribute to this gender disparity. The development of EC is closely associated with smoking and alcohol consumption, which are widely recognized as major risk factors. Smoking and drinking habits are more prevalent among men (32, 33), and long-term exposure to tobacco and alcohol can lead to chronic damage of the esophageal mucosa, thereby increasing the risk of carcinogenesis. Literature indicates that men’s lifestyle and social behavior patterns significantly impact esophageal cancer incidence. The interaction of these factors is complex and not yet fully understood, necessitating further research to elucidate the specific impact of gender on the mechanisms of esophageal cancer development (34). Elevated androgen levels in men suppress CD8+ T cell function through androgen receptor (AR) signaling, promoting T cell exhaustion and reducing anti-tumor immunity (35). Conversely, estrogen appears to confer protective effects by promoting anti-inflammatory cytokines like IL-10 and enhancing T cell-mediated immunity (36). These hormonal differences may partly explain the lower incidence and better immune-mediated tumor control observed in women. The interplay between lifestyle and endocrine factors shapes the tumor immune microenvironment and influences prognosis. For example, the immunosuppressive effects of chronic inflammation and T cell exhaustion in men may contribute to poorer outcomes and reduced responsiveness to immune-based therapies. Further research is needed to explore these mechanisms and their implications for personalized treatment strategies in locally advanced esophageal cancer.

From the patient’s perspective, age and marital status are significant factors influencing the prognosis of esophageal cancer. Age is a well-established factor influencing tumor incidence, progression, and prognosis, particularly in esophageal cancer. Previous studies have indicated that the median age at diagnosis for esophageal cancer typically falls between 65 and 75 years, with an increasing proportion of elderly patients due to the aging population. Approximately 41% of new esophageal cancer cases each year involve patients aged 75 or older (37–40). In our study, the median age at diagnosis for the included patient cohort was 71 years, which aligns with previous findings. The surge in the elderly population and the ongoing process of population aging are considered major factors contributing to the rising number of cancer-related deaths (1). Research has demonstrated that older age is associated with lower survival rates for various malignancies, including esophageal cancer (41, 42). Through optimization with X-tile software, this study identified the optimal age cutoff, further elucidating the poorer prognosis observed in elderly patients. Older patients often face challenges such as declining nutritional status, reduced immune function, and increased comorbidities, which collectively complicate treatment and reduce survival rates. Immunosenescence, defined as the gradual decline in immune system function with aging, directly impacts tumor immune surveillance and the tumor microenvironment. This process is characterized by reduced immune cell diversity, chronic low-grade inflammation (“inflammaging”), and impaired immune responses against tumors (43). As individuals age, their immune system undergoes significant remodeling, characterized by thymic involution and a decline in T cell production, resulting in reduced immune cell diversity and functionality (44). This process leads to impaired tumor immune surveillance, allowing cancer cells to evade detection and progress unchecked. Furthermore, chronic low-grade inflammation exacerbates this issue by creating a pro-tumorigenic microenvironment (45). Elevated levels of inflammatory cytokines such as IL-6 and TNF-α not only promote tumor growth but also suppress effective anti-tumor immune responses (46). These age-related immune changes contribute to both the higher incidence of esophageal cancer and the poorer prognosis observed in elderly patients. In addition to its role in tumor progression, immunosenescence significantly impacts the efficacy of immune-based therapies. Immune checkpoint inhibitors (ICIs), such as anti-PD-1/PD-L1 therapies, have revolutionized cancer treatment, including for esophageal cancer. However, their effectiveness is often reduced in elderly patients. Aging-related immune exhaustion leads to higher expression of exhaustion markers such as PD-1 and TIM-3 on CD8+ T cells, impairing their cytotoxic function (46). Moreover, the chronic inflammation associated with aging activates tumor-associated macrophages (TAMs), which further suppress anti-tumor immunity and reduce the efficacy of ICIs (47, 48). These findings highlight the need for tailored immunotherapy strategies to overcome the challenges posed by immunosenescence in elderly patients. The systemic effects of aging also complicate treatment and prognosis in elderly esophageal cancer patients. Declining nutritional status, often exacerbated by sarcopenia, reduces the ability of patients to tolerate aggressive therapies, such as surgery, chemotherapy, or radiation. Furthermore, the accumulation of senescent cells in aged tissues leads to increased levels of oxidative stress and impaired DNA repair pathways, both of which promote cancer progression and reduce the effectiveness of standard treatments (49). This underscores the complex interplay between aging, immune dysfunction, and systemic health in determining the outcomes for elderly esophageal cancer patients. Therefore, age is not only a critical prognostic factor but also a key consideration in the development of personalized treatment plans, including the potential optimization of immune-based therapies for elderly patients.

Additionally, marital status, as a social and psychological factor, is increasingly recognized for its impact on patient prognosis (50, 51). Research has shown that married patients generally receive better social support and emotional comfort compared to unmarried, divorced, or widowed patients, which helps alleviate psychological stress, enhance treatment adherence, and subsequently improve prognosis (52). Krajc et al. (53)found through a meta-analysis that married patients exhibited better overall survival and cancer-specific survival rates. Several factors contribute to the observed phenomenon in unmarried cancer patients: Economically, they often face greater financial constraints, limiting treatment options and adherence, which impacts outcomes. Lifestyle differences, including irregular sleep, poor diet, smoking, and excessive alcohol consumption, are more prevalent among unmarried patients, increasing cancer risk and undermining recovery. Additionally, the absence of a social support network exacerbates both psychological and physical health challenges, leading to feelings of loneliness, helplessness, and anxiety. Information asymmetry further complicates treatment decisions, as unmarried patients may lack professional guidance and support, affecting subsequent care.

From a tumor perspective, factors such as the primary location, size, grade, and stage (including T stage, AJCC stage, and SEER stage) are considered significant predictors of prognosis in esophageal cancer patients. Tumor grading, which directly reflects the degree of differentiation, is a key factor in determining outcomes; poorly differentiated tumors typically correlate with worse prognosis. As AJCC and SEER stages advance, tumor pathology progresses, resulting in shorter patient survival, a trend consistent with the findings of the nomogram model in this study (54). Notably, this study identified tumor location as an independent prognostic factor for esophageal cancer, particularly highlighting poor outcomes in patients with overlapping lesions of the esophagus. In comparison, patients with lower esophageal tumors had a significantly higher risk of poor prognosis than those with middle or upper esophageal tumors. These findings align with existing literature, reinforcing the strong association between tumor location and patient survival outcomes (27). This indicates that for patients with overlapping lesions, the surrounding normal esophageal tissue may have a higher propensity for malignant transformation, contributing to a poorer prognosis. From an immunological perspective, tumor location significantly influences the local immune microenvironment, which in turn impacts patient prognosis. Research has shown that tumors in the lower esophagus, predominantly EAC, often exhibit a more immunosuppressive microenvironment compared to ESCC in the middle or upper esophagus. EAC tumors frequently display higher expression of immune checkpoints such as PD-L1 and TIM-3, reduced cytotoxic T cell infiltration, and an increased presence of regulatory T cells (Tregs) and TAMs. These features collectively contribute to immune evasion and poorer responses to therapies such as chemoradiotherapy and ICIs. In contrast, ESCC tumors, located in the upper and middle esophagus, are associated with more effective immune-mediated tumor control due to higher levels of pro-inflammatory cytokines and better immune cell infiltration, aligning with their relatively better prognosis (55, 56). This suggests that EAC is more challenging to treat, leading to relatively lower survival rates. Second, differences in lymphatic drainage play a critical role in prognosis. Tumors located in the middle and upper esophagus primarily drain to the cervical and mediastinal lymph nodes, regions that are more effectively controlled by chemoradiotherapy. In contrast, lower esophageal tumors exhibit more complex lymphatic pathways, often involving multiple drainage regions in the gastric and abdominal areas. This complexity increases the risk of lymphatic metastasis and complicates treatment, leading to poorer outcomes for patients with lower esophageal tumors (57).

Tumor size is a critical indicator for assessing tumor burden and disease progression and is closely linked to prognosis across various cancer types. Larger tumors typically reflect more aggressive biological behavior, indicating faster proliferation rates and stronger invasive capabilities, which are associated with higher malignancy (58). A meta-analysis conducted by Wang et al. in 2021 demonstrated a significant association between tumor size and poorer CSS or disease-specific survival (DSS) (HR = 1.856; 95% CI: 1.173–2.937, p < 0.001), indicating that larger tumors correspond to worse prognoses (59). Larger tumors also significantly alter the immune landscape by increasing immune suppressive mechanisms. Studies suggest that larger tumors are often associated with a more immunosuppressive microenvironment, characterized by chronic antigen exposure and increased levels of immunosuppressive cytokines, which can upregulate immune checkpoint molecules such as PD-1 and LAG-3. This contributes to T-cell exhaustion and reduced cytotoxic activity over time (60–62). Additionally, larger tumors recruit more TAMs and myeloid-derived suppressor cells (MDSCs), which suppress effector T cell function and promote tumor invasion and metastasis (63, 64). These immune evasion mechanisms further explain the higher likelihood of lymph node metastasis observed in larger tumors. Tumor size is also strongly correlated with lymph node metastasis, as larger tumors are more likely to breach the basement membrane of the primary site and enter lymphatic vessels, leading to nodal involvement. Lymph node metastasis, a key marker of cancer progression, is a critical factor influencing prognosis. A meta-analysis conducted by Jiang et al. in 2021 reported that the incidence of lymph node metastasis in esophageal cancer was 24.2%, with tumor size, primary location, and degree of differentiation identified as risk factors for nodal involvement (65). Larger tumors, lower primary sites, and poorer differentiation are associated with an increased likelihood of lymph node metastasis, which in turn is indicative of a worse prognosis.

This study highlights a promising avenue for future research in the management of locally advanced unresectable esophageal cancer (LAUEC). The nomogram developed through LASSO-Cox regression provides a personalized tool for prognostic assessment, emphasizing the critical roles of chemotherapy, tumor size, and radiotherapy as key prognostic factors. The model’s validation confirms the essential role of concurrent chemoradiotherapy in improving outcomes for these patients. As the field of esophageal cancer treatment evolves, the rise of immunotherapy presents an opportunity to revolutionize current treatment paradigms, particularly for patients with locally advanced unresectable esophageal cancer. Immunotherapy, in combination with traditional chemoradiotherapy, is anticipated to significantly enhance treatment efficacy and patient outcomes. Several large-scale Phase III clinical trials are currently evaluating the efficacy of combining immunotherapy with conventional chemoradiotherapy (10). The results from these trials could provide crucial insights into integrating immunotherapy variables into prognostic models. Incorporating biomarkers such as PD-L1 expression levels, tumor mutational burden (TMB), microsatellite instability (MSI), and immune cell infiltration scores into the nomogram could significantly refine its predictive accuracy for LAUEC patients. For example, patients with high PD-L1 expression or elevated TMB often demonstrate superior responses to immunotherapy, underscoring their value as crucial prognostic variables. Integrating these biomarkers into the model could enhance its ability to stratify patients based on their likelihood of responding to novel treatment modalities. Future research should focus on incorporating these emerging variables into the existing prognostic models to further refine their accuracy and applicability. Incorporating immunotherapy-related factors could potentially offer a more comprehensive prediction of patient outcomes, reflecting the latest advancements in treatment modalities. Consequently, our prognostic model should be adjusted and optimized in light of new clinical data to offer precise and individualized guidance for clinicians.

This study has several limitations. Firstly, the lack of detailed treatment records, such as chemotherapy, immunotherapy, and targeted therapies, in the utilized database may have affected model predictive accuracy. Secondly, the retrospective nature of the SEER database used in this study may introduce selection bias, necessitating future prospective studies for validation. Additionally, the model’s applicability to non-White or regional populations is unclear, highlighting the need for external validation studies in diverse populations. Thirdly, key patient factors like height, weight, education, lifestyle habits, and laboratory/imaging data were not included due to data availability, limiting model predictive power. Future research should incorporate these variables. Finally, while the nomogram showed robust internal validation, external validation in other regions, especially Eastern populations, is required. Larger, multicenter studies are needed to confirm its global applicability and accuracy.

## 5 Conclusion

We developed and validated a personalized survival prediction nomogram for locally advanced unresectable esophageal cancer, demonstrating accurate and effective prognostic predictions. This nomogram is valuable for identifying high-risk patients and aiding in individualized treatment strategies. Its potential clinical value is underscored by its accuracy in predicting prognosis and enhancing decision-making. However, further validation in larger trials is needed. Overall, this nomogram represents a significant advancement in personalized prognostic assessment for this patient population, contributing to improved outcomes and targeted therapies. Future research should focus on validating and refining this tool.

## Data Availability

The original contributions presented in the study are included in the article/**Supplementary Material**. Further inquiries can be directed to the corresponding author.

## Supplementary material

Supplementary Figure 1
**(A–F)** The image shows defining the optimal cutoff values of age and tumor size via X-tile analysis. **(A, D)** The black dot indicates that optimal cutoff values of age/tumor size have been identified. **(B, E)** A histogram and **(C, F)** Kaplan-Meier curve were constructed based on the identified cutoff values.

Supplementary Figure 2
**(A, B)** The time-dependent ROC curves for the AJCC stage. The time-dependent ROC curves 1-, 3- and 5-year for nomogram in training set **(A)** and validation set **(B)**.
