# Supplementary material for: A nomogram for predicting cancer-specific survival in patients with locally advanced unresectable esophageal cancer: development and validation study
Source: Front Immunol. 2025 Feb 14;16:1524439. doi: 10.3389/fimmu.2025.1524439 (PMC11868048; doi:10.3389/fimmu.2025.1524439)

**Table S1 Univariable Cox regression analysis for Cancer-specific survival in the Training set**

| **Variables** | **N** | **Event N** | **HR ^a^** | **95% CI ^b^** | **p-value** |
| --- | --- | --- | --- | --- | --- |
| Sex |  |  |  |  |  |
| Female | 819 | 504 | Ref. | Ref. ^c^ |  |
| Male | 2162 | 1363 | 1.07 | 0.97, 1.18 | 0.197 |
| Age |  |  |  |  |  |
| ˂=75 | 1817 | 1129 | Ref. | Ref. |  |
| 75-85 | 868 | 521 | 1.06 | 0.96, 1.18 | 0.258 |
| ˃85 | 296 | 217 | 1.72 | 1.49, 1.99 | <0.001 |
| Race |  |  |  |  |  |
| White | 2421 | 1513 | Ref. | Ref. |  |
| Black | 345 | 225 | 1.17 | 1.02, 1.34 | 0.03 |
| Others | 215 | 129 | 0.96 | 0.80, 1.14 | 0.624 |
| Marital |  |  |  |  |  |
| Married | 1653 | 993 | Ref. | Ref. |  |
| Unmarried and others | 1328 | 874 | 1.21 | 1.10, 1.32 | <0.001 |
| Income. |  |  |  |  |  |
| ˂=70000 | 1040 | 690 | Ref. | Ref. |  |
| ˃70000 | 1941 | 1177 | 0.89 | 0.81, 0.98 | 0.016 |
| Tumor.Location |  |  |  |  |  |
| Lower | 1631 | 1057 | Ref. | Ref. |  |
| Middle | 779 | 490 | 0.93 | 0.83, 1.03 | 0.18 |
| Upper | 444 | 236 | 0.69 | 0.60, 0.79 | <0.001 |
| Overlapping | 127 | 84 | 1.11 | 0.89, 1.38 | 0.376 |
| Tumor. size |  |  |  |  |  |
| ˂=30 | 887 | 487 | Ref. | Ref. |  |
| 30-76 | 1570 | 1003 | 1.33 | 1.20, 1.49 | <0.001 |
| ˃76 | 524 | 377 | 1.79 | 1.56, 2.04 | <0.001 |
| Histology |  |  |  |  |  |
| Squamous cell carcinoma | 1465 | 856 | Ref. | Ref. |  |
| Adenocarcinoma | 1516 | 1011 | 1.22 | 1.11, 1.33 | <0.001 |
| Grade |  |  |  |  |  |
| Grade I-II | 1640 | 980 | Ref. | Ref. |  |
| Grade III-IV | 1341 | 887 | 1.27 | 1.16, 1.39 | <0.001 |
| T.stage |  |  |  |  |  |
| T1 | 710 | 430 | Ref. | Ref. |  |
| T2 | 477 | 269 | 0.72 | 0.62, 0.84 | <0.001 |
| T3 | 1518 | 968 | 0.98 | 0.87, 1.10 | 0.695 |
| T4 | 276 | 200 | 1.43 | 1.21, 1.70 | <0.001 |
| N.stage |  |  |  |  |  |
| N0 | 1417 | 853 | Ref. | Ref. |  |
| N1 | 1317 | 818 | 1.07 | 0.97, 1.18 | 0.163 |
| N2 | 212 | 166 | 1.33 | 1.12, 1.57 | <0.001 |
| N3 | 35 | 30 | 1.52 | 1.06, 2.19 | 0.024 |
| AJCC.stage |  |  |  |  |  |
| I | 606 | 365 | Ref. | Ref. |  |
| II | 675 | 380 | 0.76 | 0.66, 0.88 | <0.001 |
| III | 1379 | 882 | 1.01 | 0.89, 1.14 | 0.886 |
| IVA | 321 | 240 | 1.33 | 1.13, 1.56 | <0.001 |
| SEER Summary.Stage |  |  |  |  |  |
| Regional | 2153 | 1389 | Ref. | Ref. |  |
| Localized | 828 | 478 | 0.86 | 0.78, 0.96 | 0.005 |
| Chemotherapy |  |  |  |  |  |
| Yes | 2357 | 1399 | Ref. | Ref. |  |
| No/Unknown | 624 | 468 | 2.36 | 2.13, 2.63 | <0.001 |
| Radiotherapy |  |  |  |  |  |
| Yes | 2506 | 1517 | Ref.. | Ref. |  |
| No/Unknown | 475 | 350 | 2.3 | 2.04, 2.58 | <0.001 |

a. HR, Hazard Ratio; b.CI, Concordance Index; c. Ref., Reference;

**Table S2 The coefficients of Lasso regression analysis**

| **Variables** | **Coefficient** |
| --- | --- |
| Sex_level_Male | 0.000000000 |
| Age_level_75-85 | 0.000000000 |
| Age_level_˃85 | 0.157690738 |
| Race_level_Black | 0.000000000 |
| Race_level_Others | 0.000000000 |
| Marital_level_Unmarried and others | 0.006123039 |
| Income._level_˃70000 | 0.000000000 |
| Tumor.Location_level_Middle | 0.000000000 |
| Tumor.Location_level_Upper | -0.099704307 |
| Tumor.Location_level_Overlapping | 0.000000000 |
| Tumor.size_level_30-76 | 0.040824325 |
| Tumor.size_level_˃76 | 0.241283408 |
| Histology_level_Adenocarcinoma | 0.000000000 |
| Grade_level_GradeIII-IV | 0.078669149 |
| T.stage_level_T2 | -0.070841500 |
| T.stage_level_T3 | 0.000000000 |
| T.stage_level_T4 | 0.075702503 |
| N.stage_level_N1 | 0.000000000 |
| N.stage_level_N2 | 0.000000000 |
| N.stage_level_N3 | 0.000000000 |
| AJCC.stage_level_II | -0.013577979 |
| AJCC.stage_level_III | 0.000000000 |
| AJCC.stage_level_IVA | 0.103166629 |
| SEER.Summary.Stage_level_Localized | -0.034321361 |
| Chemotherapy_level_No/Unknown | 0.548426083 |
| Radiotherapy_level_No/Unknown | 0.336454154 |

Figure S1. (A–F) The image shows defining the optimal cutoff values of age and tumor size via X-tile analysis. (A, D) The black dot indicates that optimal cutoff values of age/tumor size have been identified. (B, E) A histogram and (C, F) Kaplan-Meier curve were constructed based on the identified cutoff values.


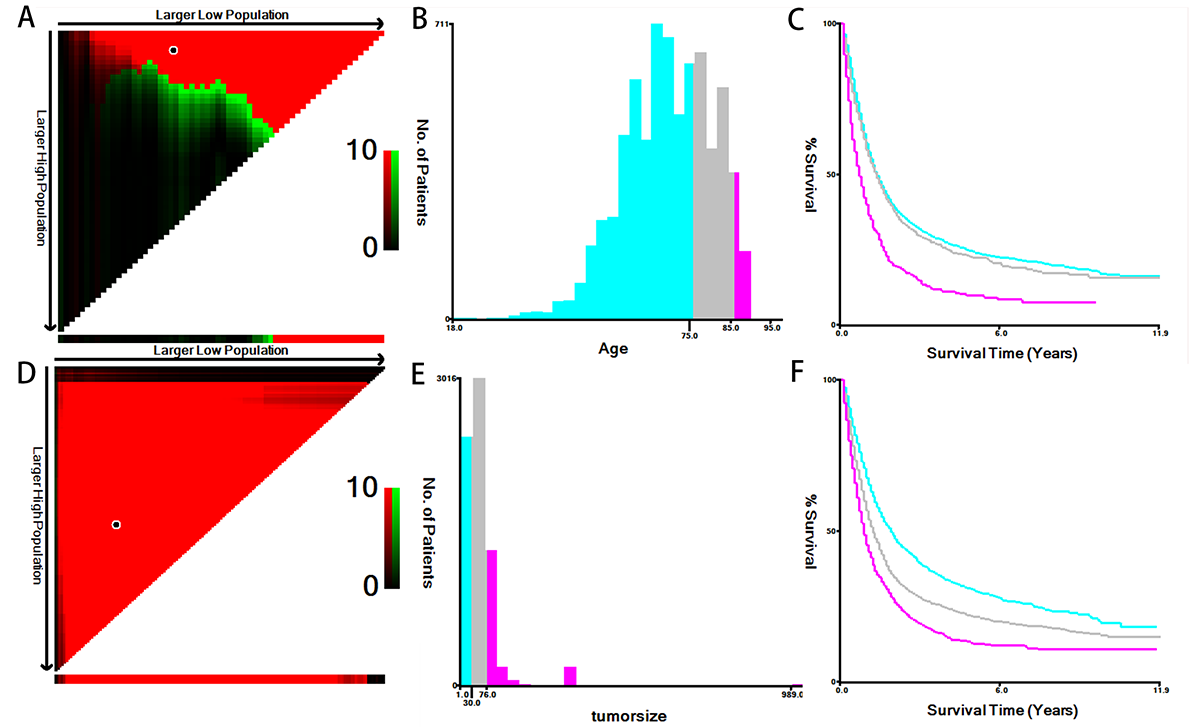


Figure S2. (A-B) The time-dependent ROC curves for the AJCC stage. The time-dependent ROC curves 1-, 3- and 5-year for nomogram in training set (A) and validation set (B).


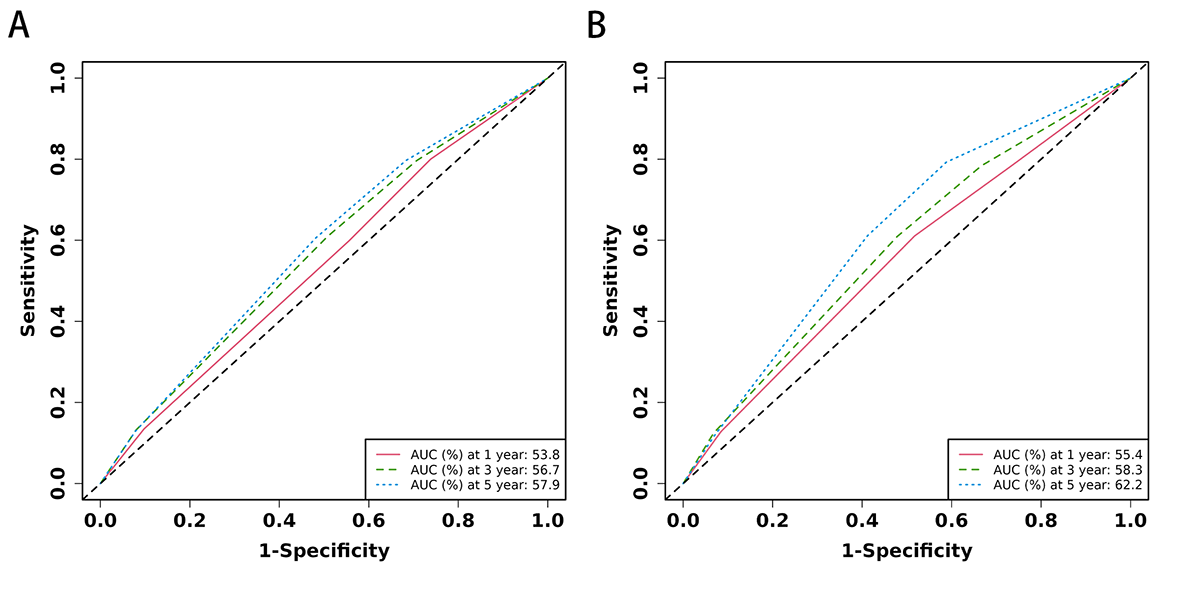

Supplement: Supplementary file 3 [file DataSheet3.docx]
